# Data on the fabrication of hybrid calix [4]arene-modified natural bentonite clay for efficient selective removal of toxic metals from wastewater at room temperature

**DOI:** 10.1016/j.dib.2021.106799

**Published:** 2021-01-30

**Authors:** Khouloud Jlassi, Kamel Eid, Mostafa H. Sliem, Aboubakr M. Abdullah, Mohamed M. Chehimi

**Affiliations:** a Centre for advanced materials, Qatar University, Doha 2713, Qatar; b Gas Processing Center, College of Engineering, Qatar University, Doha 2713, Qatar; c Univ Paris Est Creteil, CNRS, ICMPE, UMR7182, F-94320 Thiais, France

**Keywords:** Bentonite clay, Calix[4]arene, Heavy metals removal, Cd (II), Wastewater treatment

## Abstract

Fresh water resources on the earth are less than 0.2%; meanwhile, around 80% of the freshwater is consumed daily in agriculture, industries, and household activities [1], [2]. There is an essential need to develop efficient adsorbents for wastewater treatment [1], [2], [3], [4], [5], [6], in this regards, hereafter we present the rationale synthesis and characterization of hybrid natural bentonite clay modified with Calix [4] arene (denoted as B-S-Calix) as efficient adsorbents for toxic metals from wastewater. This is driven by the facile photo-radical thiol-yne addition among the thiolated clay and an alkynylated calix[4]arene. The morphology, surface modifications, and Thermal degradation of B, B-S, and B-S-Calix were investigated using TEM, FTIR, and TGA techniques. The adsorption performance of B, BS and B-S-Calix towards toxic metals including cadmium (II) ion [Cd (II)], zinc (II) ion [Zn(II)], lead(II) ion [Pb(II)], strontium(II) ion [Sr (II)], cobalt(II) ion [Co (II)], copper(II) ion [Cu(II)], and mercury (II) ion [Hg(II)] from wastewater were benchmarked 25 °C. These data are related to the article entitled “hybrid Clay/Calix[4]arene Calix[4]arene-clicked clay through thiol-yne addition for the molecular recognition and removal of Cd(II) from wastewater’’ [7].

**Specifications Table**

**Table:** 

Subject	Chemistry
Specific subject area	General chemistry
Type of data	Images
	Charts
How data were acquired	The thermal degradation was acquired using a thermogravimetric analyzer ((TGA, Perkin Elmer Pyris 1 TGA). The concentration of the removed metals was identified using Inductively coupled plasma atomic emission spectroscopy (ICP-AES). The transmission electron microscopy (TEM) images were taken by (TEM Jeol 100 CX-II, Japan). The Fourier-transform infrared (FTIR) spectra were recorded on (Nicolet Magna 860 FTIR spectroscopy (Thermo-Electron)). All the charts were drawn using Origin 2019.
Data format	TGA, TEM images, and charts for toxic metal removal are available.
Parameters for data collection	The TGA spectra were recorded under heating from 25 °C to 800 °C at a rate of 10 °C min^−1^ under an oxygen atmosphere. The TEM images were acquired after mixing with epoxy resin, then cut using a microtome. The FTIR spectra were measured using KBr compressed pellets with the samples with a weight ratio of 3/1.
Description of data collection	The fabrication process, thermal degradation, morphology, and adsorption performance using B, B-S, and B-S-Calix towards toxic Cd (II), Zn(II), Pb(II), Sr(II), Co(II), Cu(II), and Hg(II) removal, was measured at 25 °C.
data source location	Center for advanced materials, qatar university, doha, qatar.
Data accessibility	The raw data were introduced in the form of Microsoft Excel file.
Related research article	K. Jlassi K. K. Eid, M.H. Sliem, A. M. Abdullaha, M. M. Chehimi, Calix[4]arene-clicked clay through thiol‑yne addition for the molecular recognition and removal of Cd(II) from wastewater, Separation and Purification Technology 251 (2020) 117383. https://doi.org/10.1016/j.seppur.2020.117383[7].
	

## Value of the Data

• Fabrication of modified bentonite-based adsorbents is important in wastewater treatment applications.
• Calix[4]arene-modified bentonite clay was obtained by photo-radical-mediated thiol-yne addition.
• The data herein are useful for scientists, environmental engineers, and industrial end-users.
• The data can be used for developing effective and low-cost natural-based adsorbents for water treatment.

## 1 Data Description

**Fig. 1:**
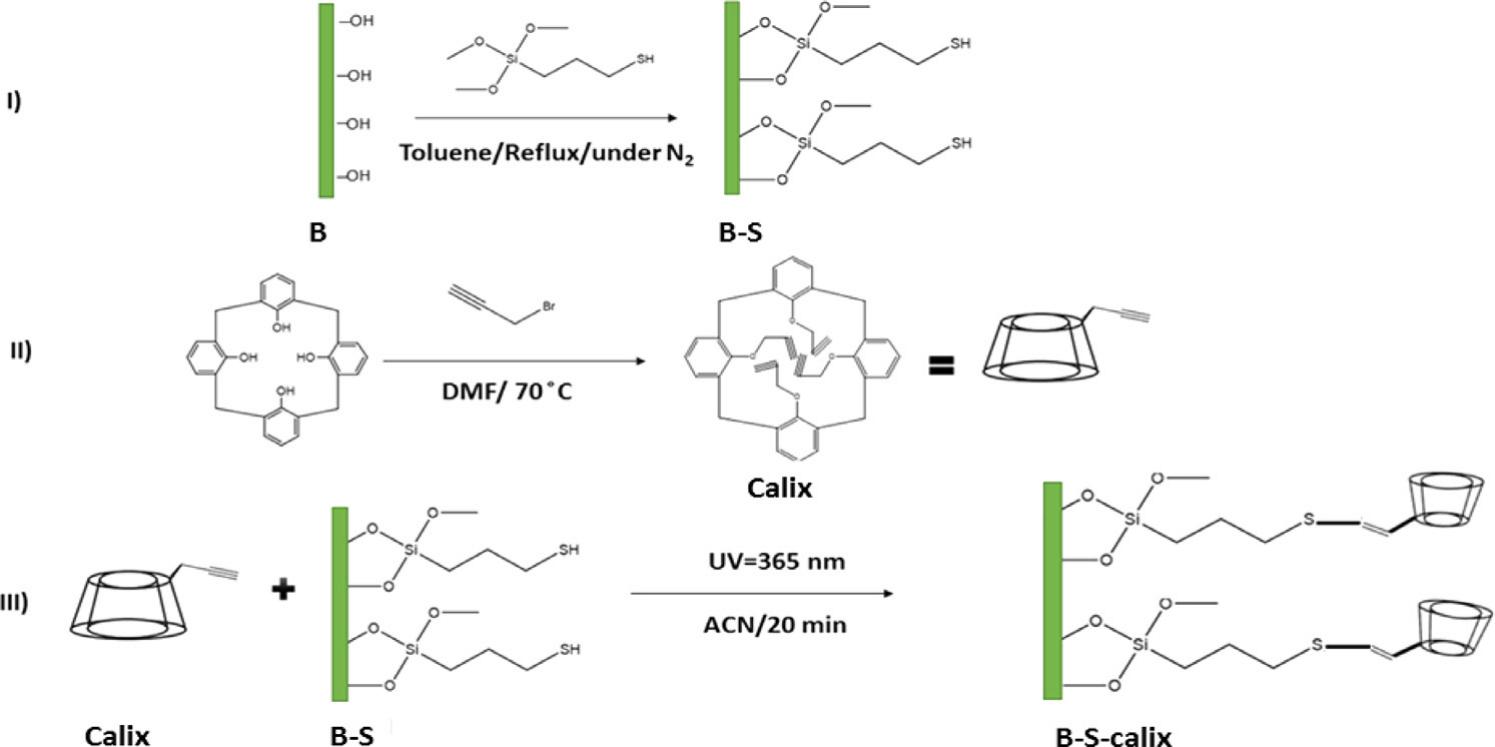
The main steps for the fabrication of B-S-Calix including (I) silanization of clay (B-S), (II) formation of calix[4] arene with a triple bond (Calix), and finally (III) hybrid B-S-Calix, this image is readapted from the related research article [7].

**Fig. 2:**
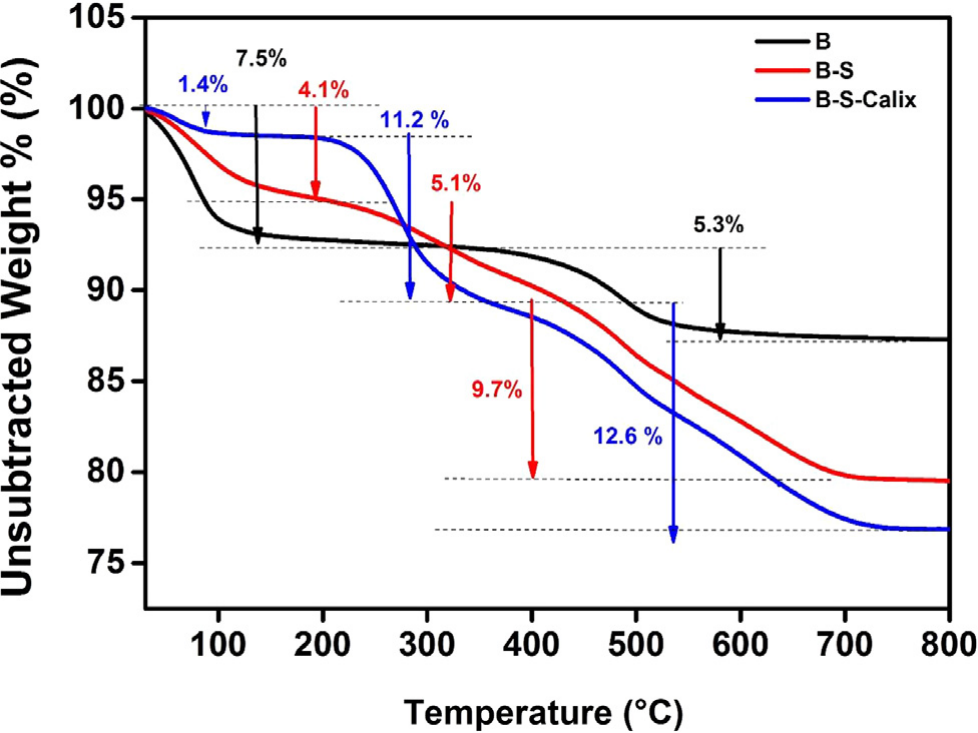
TGA patterns of B, B-S and B-S-calix and modified bentonite.

**Fig. 3:**
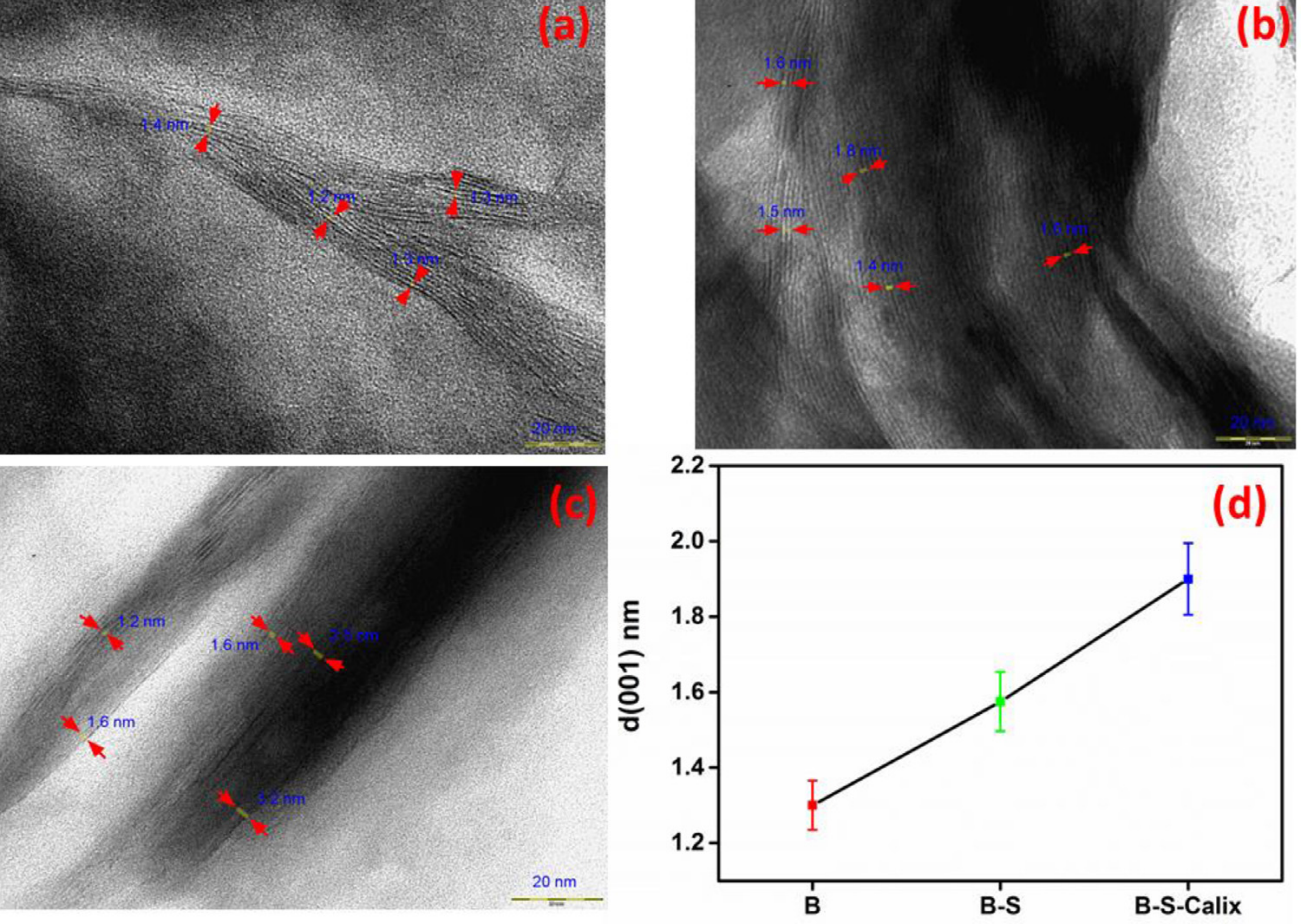
(a,b, and c) high-resolution TEM images of B, B-S, and B-S-Calix, (d) Evolution of basal distance d (001), in the presence of the differently prepared samples.

**Fig. 4:**
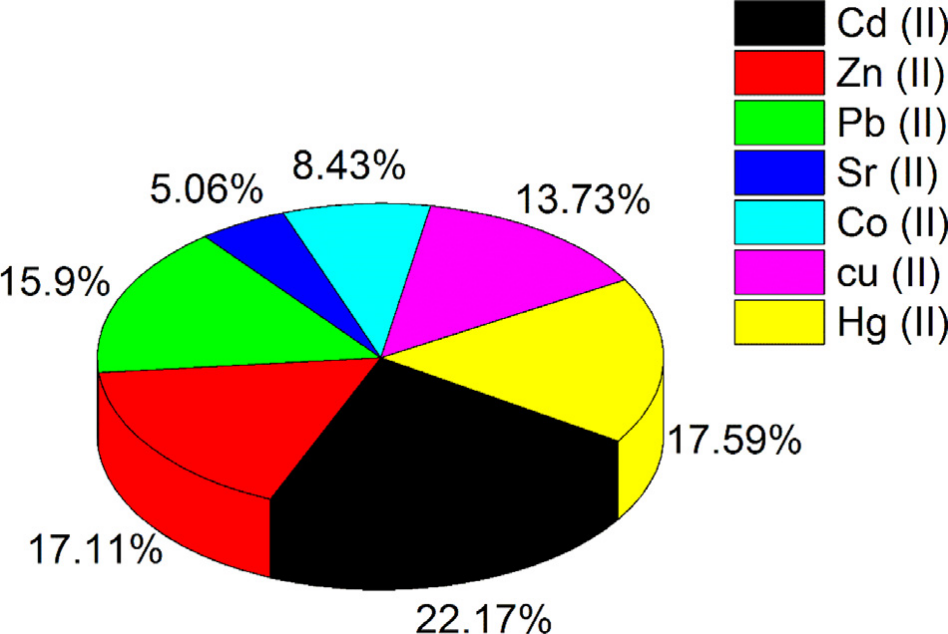
The selective adsorption of Cd(II) on B-S-Calix in the presence of Zn(II), Pb(II), Sr (II), Co(II), Cu(II), and Hg (II) toxic metals.

This article presents the data associated with the modification of bentonite clay (B), silanized bentonite (B-S) with Calix[4]arene (B-S-Calix), and its utilization to remove toxic metals from wastewater [7]. The data are the synthesis of B-S-Calix, including the initial silanization of bentonite clay with mercaptosilane (B-S), then fabrication of 5,11,17,23-tetra-t-butyl-25,26-bis(O-propargyl) calix[4]arene, followed by its combination with clay to form (B-S-Calix) (Fig. 1) in addition to the TGA (Fig. 2), TEM images (Fig. 3a-c of B, B-S, and B-S-Calix, respectively, and the basal distance for all samples (Fig. 3d). Also, the percentage of surface modification organic moieties in B-S and B-S-Calix is illustrated in Table 1. Besides, the utilization of B-S-Calix for selective removal of Cd(II) from wastewater in the presence of Zn(II), Pb(II), Sr(II), Co(II), Cu(II), and Hg(II) is presented in Fig. 4. Raw data are provided as supplementary files.

**Table 1:** The amount of intercalated organic moieties deduced from the TGA experiments for modified clays.

Samples	Intercalated moieties (%) by weigh in the sample
B-S	16.3
B-S-Calix	22.3

## 2 Experimental Design, Materials and Methods

### 2.1 Preparation process of B-S-Calix

All Chemicals used here were purchased from Sigma-Aldrich (Taufkirchen, Germany). Fig. 1 shows the fabrication process of B-S-Calix using the photo-induced thiol-yne method. This process includes three main steps: First, intercalating the sodium-modified bentonite clay by the 3-mercaptopropyltrimethoxysilane to form silanized clay (denoted as B-S), followed by the preparation of calix[4] arene with triple bond via mixing a solution of 5,11,17,23-tetra-tbutylcalix[4]arene, propargyl bromide in dimethylformamide, and sodium hydride at 80 °C to produce calix[4] arene with a triple bond (denoted as Claix), and finally, the photo-induced thiol-yne method under UV-light (365 nm) illumination for the silanized clay (B-S) with Calix dissolved in acetonitrile solution (CAN) to form the B-S-Calix.

Fig. 2 demonstrates the TGA curves of B, B-S, and B-S-Calix. The corresponding percentage of organic moieties (silane coupling agent and calixarene) was summarized in Table 1. The B-S and B-S-Calix-modified clays have shown a noteworthy improvement in thermal stability compared to the unmodified B. For the purified B sample, a mass loss of 7.5% at 40–150 °C was noted; moreover, a 5.3% weight loss appeared at 400–800 °C, for the -OH groups desorption from the bentonite surface. For B-S and B-S-Calix clays, the highest weight loss was noted at 200–650 °C, which is related to the degradation of organic compounds (silane coupling and calixarene). This thermal degradation of 5.7% for B-S and 11% for B-S-Calix in the 1st step of calix bonded to clay via Van der Waals forces, alongside a 9.7% for B-S and 12.6% for B-S-Calix in the 2nd step of calix bonded to bentonite of (Fig. 2) [8,9].

Compared to bentonite, the formation of B-S and B-S-Calix was confirmed by the FTIR analysis using KBr compressed pellets, from 400–4000 cm^−1^ as deeply described in the main article [7]. The surface area of the adsorbent is of great importance in the adsorption process. Thereby, the surface area of B-S-Calix and bentonite were calculated to form the nitrogen adsorption-desorption isotherm curves using the Brunauer–Emmett–Teller (BET) model [7]. The determined BET surface area of B-S-Calix (78.934 m^2^/g) was higher than that of bentonite (15.75 m^2^/g). Additionally, the particle size B-S-Calix was about 140 ± 10 nm and had a negative charge of (−36.05 mV) on the surface as determined by the DLS and zeta potential, respectively [7].

The shape of the as-obtained materials was analyzed by TEM (Fig. 3). The TEM images of B (Fig. 3a), B-S (Fig. 3b), and B-S-Calix (Fig. 3c) show the multilayered structure. However, B and B-S were slightly compacted and more staked than that of B-S-Calix. The layers of B-S-Calix were intercalated and in some regions exfoliated. Fig. 3d shows the increase of basal distances, between bentonite layers d(001) for each sample, upon sample modifications, the d(001) deduced from the TEM images, were calculated through some selected regions, in our case four regions were selected. For B-S and especially for B-S-Calix some regions are intercalated and some others are slightly exfoliated, the average basal distances between layers deduced from TEM were found to be equal to 1.3, 1.5 and 1.9 nm, for B, B-S, and B-S-Calix respectively, which is slightly higher, but in the same range and match the distances determined by TEM and XRD described in the main related manuscript [7], demonstrating successful intercalation of the calixarene via thiol-yne click reaction.

The selectivity of B-S-Calix toward the adsorption of Cd (II) was tested using Zn (II), Pb(II), Sr (II), Co(II), Cu(II), and Hg (II) toxic metals under pH of 8 at 25 oC (Fig. 4). These overall toxic metals are highly dangerous to human and animal health [1,^5^,[10], [11], [12], [13], [14], [15]. A dispersion of B-S-Calix in wastewater solution contains 50 ppm of each heavy metal under stirring for 1 h followed by the phase separation and then analyzing the supernatant using ICP.

The adsorption efficiency (E%) was determined using the following equation

(1) $$E=\left[\left(C_{f}-C_{o}/C_{o}\right)\times 100\right]$$

Where C_f_ is the final concertation of metals in the supernatant and C_o_ is the initials metal concentration. The metals adsorption ability on B-S-Calix was found to be around 92, 73, 72, 66, 57, 35, and 21% for Cd(II), Hg(II), Zn(II), Pb(II), Cu(II), Co(II), and Sr (II), respectively (Fig. 4).

## 3 Feasibility for the Large-Scale Water Treatment

The cost of adsorbents is a highly important factor, determine the feasibility for large-scale applications. Based on the cost of the raw materials used in the preparation of B-S-Calix, its production cost was estimated to be around 0.1 ± 0.03 $ USD per one gram, which is more cost-effective than most of the commercial adsorbents such as carbon and silica. Meanwhile, our developed C-S-Calix's water treatment efficiency was significantly higher than most previous adsorbents under similar conditions (Table 4 in the related manuscript) [7].

Considering the initial concentration of 50 ppm in the contaminated water, B-S-Calix removed 92.8% of Cd(II), so the remaining Cd(II) is 3.6 ppm, which is higher than the maximum contaminant level of Cd in drinking water (0.005 ppm) according to the World Health Organization, so the produced water is not feasible for drinking but can be used for agriculture usage. On the other hand, although the Cd(II) removal percentage over B-S-Claix was only 92.8% at pH of 8, at lower concertation of Cd(II) (25 ppm), the removal effeciency was 100% under the same conditions.

## CRediT Author Statement

**Khouloud Jlassi:** Conceptualization, Methodology; **Kamel Eid:** Writing - original draft; **Mostafa H. Sliem:** Characterizations, Investigation; **Aboubakr M. Abdullah:** Supervision, Visualization, Validation; **Mohamed M. Chehimi:** Writing-review & editing.

## Declaration of Competing Interest

The authors declare no competing interests.

## Appendix Supplementary materials

Image, application 1
